# Effect of a Wearable Device–Based Physical Activity Intervention in North Korean Refugees: Pilot Randomized Controlled Trial

**DOI:** 10.2196/45975

**Published:** 2023-07-19

**Authors:** Ji Yoon Kim, Kyoung Jin Kim, Kyeong Jin Kim, Jimi Choi, Jinhee Seo, Jung-Been Lee, Jae Hyun Bae, Nam Hoon Kim, Hee Young Kim, Soo-Kyung Lee, Sin Gon Kim

**Affiliations:** 1 Division of Endocrinology and Metabolism Department of Internal Medicine Korea University College of Medicine Seoul Republic of Korea; 2 Division of Endocrinology and Metabolism Department of Medicine Samsung Medical Center, Sungkyunkwan University School of Medicine Seoul Republic of Korea; 3 Department of Food and Nutrition Inha University Incheon Republic of Korea; 4 Division of Computer Science and Engineering Sun Moon University Asan Republic of Korea

**Keywords:** digital health intervention, wearable activity tracker, physical activity intervention, Fitbit, North Korean refugees, metabolic risk management, step counts

## Abstract

**Background:**

Effective health interventions for North Korean refugees vulnerable to metabolic disorders are currently unelucidated.

**Objective:**

This study aimed to evaluate the effects of digital health interventions in North Korean refugees using a wearable activity tracker (Fitbit device).

**Methods:**

We conducted a prospective, randomized, open-label study on North Korean refugees aged 19-59 years between June 2020 and October 2021 with a 12-week follow-up period. The participants were randomly assigned to either an intervention group or a control group in a 1:1 ratio. The intervention group received individualized health counseling based on Fitbit data every 4 weeks, whereas the control group wore the Fitbit device but did not receive individualized counseling. The primary and secondary outcomes were the change in the mean daily step count and changes in the metabolic parameters, respectively.

**Results:**

The trial was completed by 52 North Korean refugees, of whom 27 and 25 were in the intervention and control groups, respectively. The mean age was 43 (SD 10) years, and 41 (78.8%) participants were women. Most participants (44/52, 95.7%) had a low socioeconomic status. After the intervention, the daily step count in the intervention group increased, whereas that in the control group decreased. However, there were no significant differences between the 2 groups (+83 and –521 steps in the intervention and control groups, respectively; *P*=.500). The effects of the intervention were more prominent in the participants with a lower-than-average daily step count at baseline (<11,667 steps/day). After the 12-week study period, 85.7% (12/14) and 46.7% (7/15) of the participants in the intervention and control groups, respectively, had an increased daily step count (*P*=.05). The intervention prevented the worsening of the metabolic parameters, including BMI, waist circumference, fasting blood glucose level, and glycated hemoglobin level, during the study period.

**Conclusions:**

The wearable device–based physical activity intervention did not significantly increase the average daily step count in the North Korean refugees in this study. However, the intervention was effective among the North Korean refugees with a lower-than-average daily step count; therefore, a large-scale, long-term study of this intervention type in an underserved population is warranted.

**Trial Registration:**

Clinical Research Information Service KCT0007999; https://cris.nih.go.kr/cris/search/detailSearch.do/23622

## Introduction

### Background

Metabolic syndrome (MetS) is characterized by abdominal obesity, insulin resistance, hypertension, and dyslipidemia, which usually reflect overnutrition and excess adiposity [1]. MetS is associated with cardiovascular disease and has become a major health hazard in the modern world, therefore, it should be prevented and managed properly [1-3]. Among the various determinants of MetS, lifestyle is an environmental factor that plays a critical role in exacerbating or improving metabolic health [4]. Individuals who immigrate from low-income to high-income countries are more likely to experience drastic changes in their lifestyle, including changes in dietary habits, unusual light-dark cycles, and other stressful changes [5]. Epidemiological studies have demonstrated the negative effect of immigration on the metabolic profile, with the increasing prevalence of chronic diseases in immigrant populations [6].

North Korea and South Korea, formerly a single nation, have been separated for >70 years, leading to dramatically different social, economic, and political environments. Although South Korea has achieved rapid economic growth**,** North Korea has been unable to meet the basic needs of its impoverished population [7]. Many starving North Koreans have left North Korea and resettled in other countries, including South Korea [8]. A recent study that included approximately 900 North Korean refugees reported that excess weight gain after immigration from North Korea increased the risk of MetS, emphasizing the importance of this unique population maintaining a healthy lifestyle [9]. However, lifestyle monitoring and interventions can be challenging for immigrant populations because of their low socioeconomic status (SES) and barriers to accessing health care [10].

A wearable activity tracker enables users to monitor their activity and modify their behavior toward a healthier lifestyle [11,12]. This electronic device is typically worn on the wrist to track daily activity through parameters such as step count, calories burned, heart rate, and sleep patterns [11,13]. While being worn by users, it can provide continuous health-related information in real time. Furthermore, it can measure lifestyle-related factors without the need for observing users, which can be an effective strategy for managing MetS, especially in vulnerable people, including those who are reluctant to visit health care facilities. Nevertheless, few trials have evaluated wearable devices as a tool for assessing the lifestyle of refugees.

### Objectives

This study aimed to evaluate the effects of a digital health intervention in North Korean refugees using the wearable activity tracker Fitbit Charge 3 (Fitbit). We investigated whether this personalized digital health care intervention would increase the daily step count and improve the metabolic parameters of North Korean refugees settled in South Korea.

## Methods

### Trial Design and Participants

This was a prospective, randomized, open-label, parallel-group, single-center pilot study. The trial was conducted at the tertiary medical center of Korea University Anam Hospital. Through the Hana Center, a representative welfare center for North Korean refugees supported by the South Korean government, we recruited North Korean refugee volunteers aged 19-59 years who were able to use smartphones. The volunteers visited Korea University Anam Hospital and were screened for eligibility.

**Figure 1:**
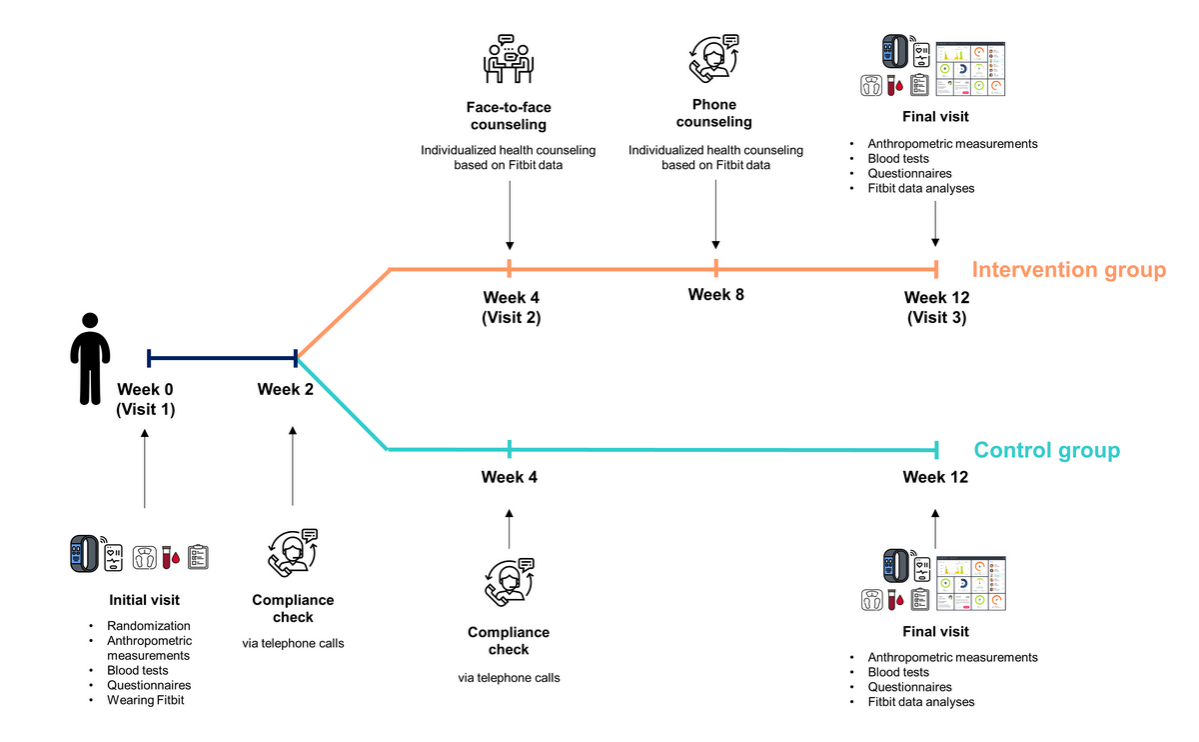
Flow diagram of the study design.

A flow diagram of the study design is shown in Figure 1. The trial protocol is provided in Multimedia Appendix 1 and is registered with the Clinical Research Information Service (CRIS KCT0007999).

At the first visit (visit 1), all participants underwent anthropometric measurements, including height, weight, and waist circumference (WC) measurements; blood pressure measurements; and blood tests, including the fasting blood sugar (FBS), glycated hemoglobin (HbA_1c_), total cholesterol, triglyceride (TG), and high-density-lipoprotein cholesterol (HDL-C) tests, after fasting overnight. The participants completed a questionnaire regarding their medical and social histories, including occupation, family income, and duration of residence in South Korea and North Korea. SES, estimated by comparing their family income with the 2021 national income deciles provided by the Korean Statistical Information Service [14], was classified into 3 groups: low (1st to 3rd deciles), moderate (4th to 7th deciles), and high (8th to 10th deciles). A questionnaire on health-related lifestyle factors, including smoking, drinking, dietary, and physical activity habits, was administered. The Korean version of the Center for Epidemiological Studies Depression Scale (CES-D) was used to measure depressive symptoms. A CES-D score of ≥16 indicated a depressed mood [15,16].

The Fitbit Charge 3, along with the corresponding application, was provided to all participants during the first visit. The participants were taught how to use the Fitbit device and were asked to wear it continuously until the end of the study. Compliance with wearing the Fitbit device was checked at weeks 2 and 4.

We divided the participants into 2 groups: an intervention group (who wore the Fitbit device and received individualized health counseling based on the Fitbit data) and a control group (who wore the Fitbit device but did not receive individualized counseling). Block randomization was performed in a 1:1 ratio to stratify the participants according to age and sex. The intervention group visited the hospital during week 4 (visit 2) and received a daily record of the step count, sleep duration, and calorie consumption. On the basis of these Fitbit data, a medical professor of endocrinology provided individualized counseling on physical activity and recommended the type, intensity, and frequency of exercise. At week 8, the intervention group received additional health counseling based on Fitbit data via telephone calls.

The end-of-trial visit was conducted at week 12 (visit 3), and both groups underwent anthropometric measurements, blood pressure measurements, and laboratory assessments. They were administered the same questionnaire regarding dietary habits and physical activity as that administered during the first visit. The Fitbit data collected over 12 weeks were used in the analyses.

### Primary and Secondary Outcomes

The primary outcome was the change in the average daily step count. The average daily step count was calculated as the mean step count throughout a specific week during the study period. The step count of visit 1 was defined as the mean number of steps during the first week after the initial visit (days 1-7). The step counts of visits 2 and 3 were defined as the mean step counts during the week immediately before the second and third visits, respectively (days 22-28 and days 78-84, respectively). The secondary outcome was the changes in the metabolic parameters, including systolic blood pressure, diastolic blood pressure, body weight, BMI, WC, FBS level, HbA_1c_ level, TG level, and HDL-C level. The proportion of patients with MetS was evaluated using the Adult Treatment Panel III guidelines [17] and WC cutoff points for the Asian population set by the World Health Organization [18]. Thus, the participants who met 3 or more of the following criteria were classified as having MetS: WC ≥90 cm in men and ≥80 cm in women, serum TG level ≥150 mg/dL, HDL-C level <40 mg/dL in men and <50 mg/dL in women, FBS level ≥100 mg/dL or recipient of antidiabetic treatment, and blood pressure ≥130/85 mm Hg or recipient of antihypertensive treatment.

### Statistical Analysis

Continuous data are presented as the mean (SD) or as the median (IQR) and were compared using the Student *t* test or the Wilcoxon rank-sum test. Categorical data are presented as the number (%) and were compared using the chi-square or Fisher exact test. The change in the average daily step count was calculated by subtracting the mean step count at visit 1 from the mean step count at a specific visit (visit 2 or 3). The percent change in the average daily step count was calculated as 100×([mean step count at a specific visit-mean step count at visit 1]/mean step count at visit 1). A mixed model for repeated measures was used to compare the changes between the intervention and control groups. The numbers (%) of participants with a higher average daily step count at visit 3 than at visit 1 were also compared using the chi-square or Fisher exact test. The changes in the metabolic parameters were first analyzed in each group by comparing the parameters at the beginning of the trial (visit 1) with those at the end of the trial (visit 3). The comparisons were performed using the paired *t* test or Wilcoxon signed rank test. Next, the changes in the parameters were compared between the intervention and control groups using the Student *t* test. The proportions of participants with MetS were also compared between the 2 groups. Additionally, the proportions of participants demonstrating improved metabolic outcomes (decreases in the systolic blood pressure, diastolic blood pressure, body weight, BMI, WC, FBS level, HbA_1c_ level, and TG level and increase in the HDL-C level) were compared between the 2 groups using the chi-square test.

Given that the intervention effect may differ according to the baseline step count, we divided the participants into 3 groups according to the average daily step count at visit 1 (>15,000, 10,000-15,000, and <10,000 steps/day) and tracked the step count until the final visit. Subgroup analyses were performed for the participants with a lower-than-average daily step count at visit 1 (<11,667 steps/day), considering that the intervention might have had a minimal effect on the participants with a high step count at baseline. To evaluate the effects of walking, we compared the changes in the metabolic parameters between the participants with and without an increased step count during the study period.

All statistical analyses were performed using the SAS software (version 9.4; SAS Institute, Inc) and the R software (version 4.1.1; R Foundation for Statistical Computing). Statistical significance was determined using a 2-sided *P* value of <.05.

### Ethics Approval

This study was approved by the institutional review board (IRB) of Korea University Anam Hospital (2020AN0079). The trial was conducted in accordance with the principles of the Declaration of Helsinki. Prior to participation, all subjects were thoroughly informed about the objectives and methodology of the research and provided written informed consent. For those participating in secondary data analysis, it is important to note that the original informed consent documents, approved by the relevant IRB, permitted the use of this data for subsequent research purposes without requiring additional consent. Considering that they are a vulnerable population, participants’ privacy and confidentiality were of the utmost importance throughout the research process. All data collected were anonymized and deidentified prior to analysis to protect the identities of the participants. Any information that could potentially identify individuals was securely stored and only accessible to the primary research team. In terms of compensation, participants in the primary research were compensated with monetary payment. The amount was deemed appropriate and ethical by the Korea University Anam Hospital Ethical Committee, considering the time commitment and contributions made by the participants. The compensation was not contingent on the participants’ responses, and they were informed that they could withdraw from the study at any time without penalty.

## Results

### Participant Characteristics

We screened 83 adults for eligibility, among whom 77 were randomly assigned to the intervention or control group (Figure S1 in Multimedia Appendix 2). The first patient was enrolled on June 13, 2020, and the last patient was enrolled on July 10, 2021. Of these 77 patients, 22 withdrew their consent, and 3 were lost to follow-up. Thus, 52 participants completed the study, of whom 27 and 25 were in the intervention and control groups, respectively. At visit 1, the Fitbit data of 2 participants in the intervention group were lost because of low compliance. Thus, the primary outcome was analyzed using data from 50 participants (25 per group). Compliance rates with wearing the Fitbit device during the entire study period (ie, the percentage of days when heart rates were observed for more than two-third of the daytime hours during the study period) in the intervention and control groups were 95.4% and 89%, respectively.

The baseline characteristics of the intervention and control groups did not differ significantly (Table 1). The mean age of the participants was 43 years, and 78.8% (41/52) were women. Of the participants, 21.1% (11/52) engaged in vigorous-intensity physical activity for ≥3 days per week. Most participants (44/52, 95.7%) had a low SES. Half (26/52, 50%) of the participants were unemployed, and 19.2% (10/52) were students. The remaining participants had varied jobs and included restaurant workers, machine workers, wiremen, and facility managers (Table S1 in Multimedia Appendix 2). Depressed mood, as indicated by a CES-D score of ≥16, was observed in 46.2% (24/52) of the participants. The median duration of residence in South Korea was 2.8 (IQR 1.2-9.7) years. The mean BMI, WC, and HbA_1c_ level of the participants were 23.2 kg/m^2^, 80.2 cm, and 5.4%, respectively, and 13.5% (7/52) of the participants had MetS.

**Table 1:** Baseline characteristics of the study participants.

			All	Intervention	Control	*P* value^a^	
Total participants, n			52	27	25		
Age (years), mean (SD)			43.2 (10.0)	43.4 (10.2)	43.0 (10.0)	.86	
Female sex, n (%)			41 (78.8)	22 (81.5)	19 (76.0)	.63	
Current drinker, n (%)			32 (64.0)	15 (57.7)	17 (70.8)	.33	
Current smoker, n (%)			10 (20.0)	5 (19.2)	5 (20.8)	.89	
**Physical activity of vigorous intensity, n (%)**							.72
	Never	29 (55.8)		16 (59.3)	13 (52.0)		
	<3 days/week	12 (23.1)		5 (18.5)	7 (28.0)		
	≥3 days/week	11 (21.1)		6 (22.2)	5 (20.0)		
**Socioeconomic status, n (%)**							.49
	Low	44 (95.7)		23 (92.0)	21 (100.0)		
	Middle	2 (4.3)		2 (8.0)	0 (0.0)		
	High	0 (0.0)		0 (0.0)	0 (0.0)		
**Occupation, n (%)**							>.99
	Permanent worker	5 (9.6)		3 (11.1)	2 (8.0)		
	Temporary worker	7 (13.5)		4 (14.8)	3 (12.0)		
	Student	10 (19.2)		5 (18.5)	5 (20.0)		
	Unemployed	26 (50.0)		13 (48.1)	13 (52.0)		
	Others	4 (7.7)		2 (7.4)	2 (8.0)		
Duration of residence in South Korea (years), median (IQR)			2.8 (1.2-9.7)	3.1 (1.4-10.3)	2.7 (1.0-8.7)	.26	
Metabolic syndrome, n (%)			7 (13.5)	4 (14.8)	3 (12.0)	>.99	
SBP^b^ (mm Hg), mean (SD)			116.2 (10.4)	117.7 (15.9)	116.2 (10.4)	.69	
DBP^c^ (mm Hg), mean (SD)			72.9 (11.9)	75.4 (11.3)	72.9 (11.9)	.44	
Height (cm), mean (SD)			157.3 (6.8)	156.0 (6.6)	158.6 (6.9)	.16	
Body weight (kg), mean (SD)			57.4 (7.2)	56.8 (8.1)	58.1 (6.2)	.52	
BMI (kg/m^2^), mean (SD)			23.2 (2.3)	23.3 (2.8)	23.0 (1.8)	.66	
WC^d^ (cm), mean (SD)			80.2 (7.9)	79.9 (9.6)	80.5 (5.6)	.75	
FBS^e^ (mg/dL), mean (SD)			97.2 (8.8)	100.6 (12.5)	97.2 (8.8)	.25	
HbA_1c_^f^ (%), mean (SD)			5.4 (0.6)	5.5 (0.7)	5.3 (0.4)	.26	
Total cholesterol (mg/dL), mean (SD)			191.3 (35.4)	191.4 (40.4)	191.2 (29.8)	.98	
TG^g^ (mg/dL), median (IQR)			80.5 (58.0-103.5)	82.0 (62.0-103.5)	75.0 (56.0-101.0)	.88	
HDL-C^h^ (mg/dL), mean (SD)			56.1 (11.0)	58.2 (11.4)	56.1 (11.0)	.51	
LDL-C^i^ (mg/dL), mean (SD)			113.1 (34.7)	109.2 (41.2)	117.2 (26.2)	.40	
25(OH)VitD^j^ (ng/dL), mean (SD)			17.3 (7.1)	16.6 (6.1)	18.1 (8.0)	.45	
CES-D^k^ score, mean (SD)			19.0 (12.8)	20.3 (13.2)	17.6 (12.6)	.45	
CES-D score ≥16, n (%)			24 (46.2)	14 (51.9)	10 (40.0)	.39	

^a^*P* value calculated using Student’s *t* test or the Wilcoxon rank-sum test for continuous variables and using the chi-square test or Fisher exact test for categorical variables.

^b^SBP: systolic blood pressure.

^c^DBP: diastolic blood pressure.

^d^WC: waist circumference.

^e^FBS: fasting blood sugar.

^f^HbA_1c_: glycated hemoglobin.

^g^TG: triglyceride.

^h^HDL-C: high-density-lipoprotein cholesterol.

^i^LDL-C: low-density-lipoprotein cholesterol (LDL-C=Total cholesterol–HDL-5/TG).

^j^25(OH)VitD: 25-hydroxyvitamin D.

^k^CES-D: Center for Epidemiological Studies Depression Scale.

### Changes in the Average Daily Step Count

**Figure 2:**
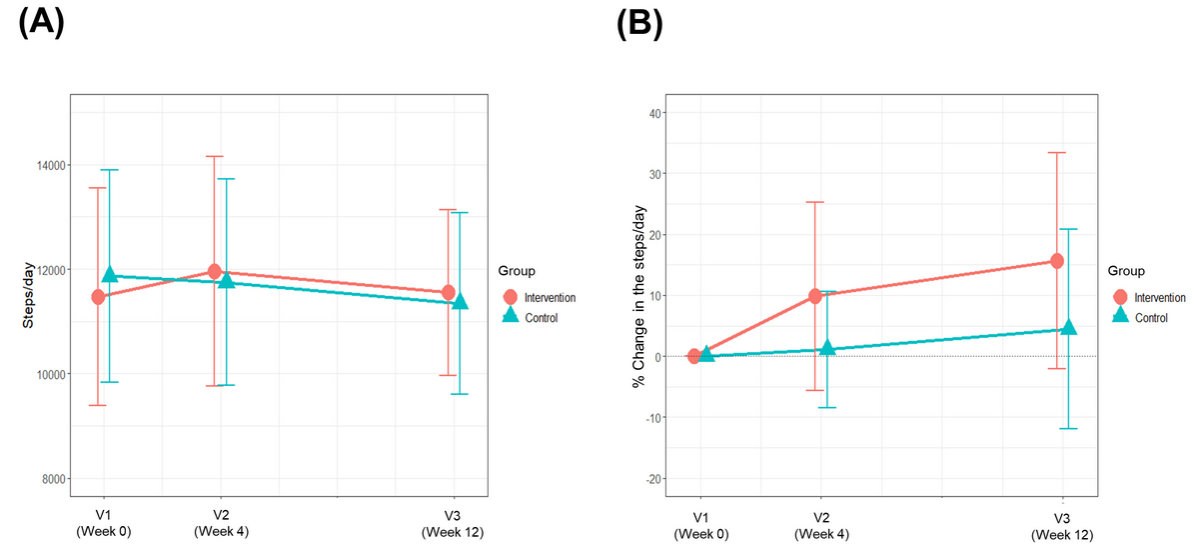
Changes in the step count during the study period for the intervention and control groups. (A) Average daily step counts. (B) Percent changes in the average daily step counts. V: visit.

Table 2 and Figure 2 show the changes in the average daily step counts in the 2 groups during the study period. The baseline average daily step counts in the intervention and control groups were 11,467 and 11,866, respectively (*P*=.76). The change in the average daily step count between visits 1 and 3 in the intervention group was +83 steps, whereas that in the control group was –521 steps (*P*=.50). The percent change in the daily step count in the intervention group was +15.7%, whereas that in the control group was +4.4% (*P*=.20). At the end of the trial, 64% (16/25) and 40% (10/25) of the participants in the intervention and control groups, respectively, had an increased daily step count; however, the difference between the 2 groups was not statistically significant (*P*=.09).

**Figure 3:**
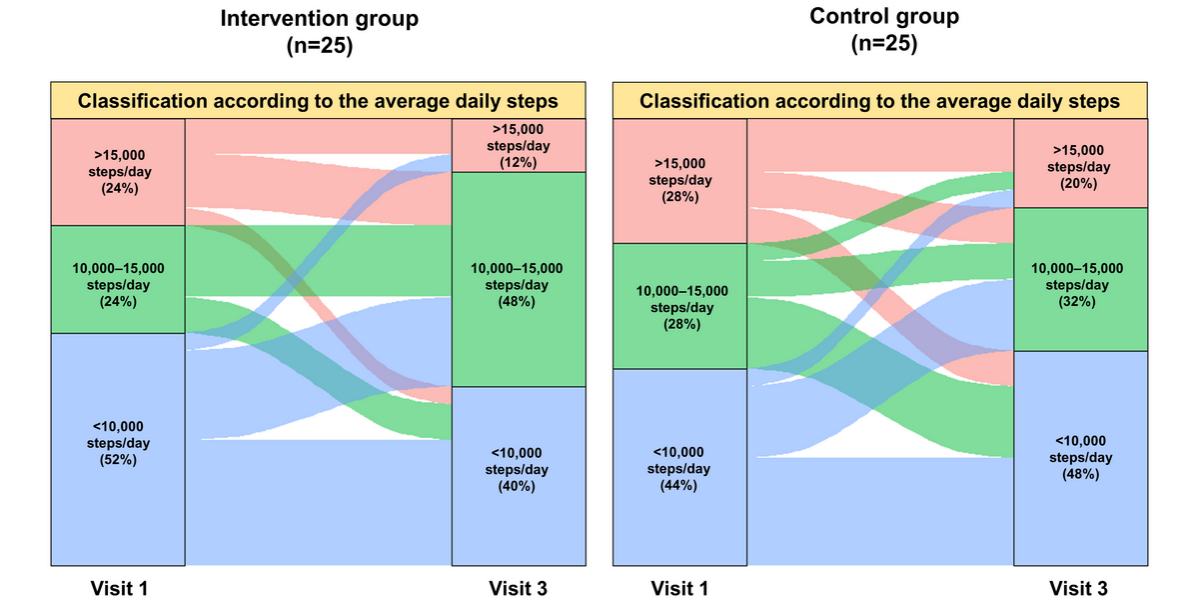
Changes in the average daily step count according to the baseline average daily step count.

The change in the average daily step count according to the baseline average daily step count is presented in Figure 3. In both groups, approximately half of the participants walked >10,000 steps per day at baseline (12/25, 48% in the intervention group and 14/25, 56% in the control group). The change in the average daily step count differed according to the baseline average daily step count, and the increase in the step count was more prominent in the participants who walked <10,000 steps/day. Only the participants with a lower-than-average daily step count at baseline (<11,667 steps/day) were included in the subgroup analyses, and the step count increased more in the intervention group than in the control group (Table 2). In total, 85.7% (12/14) and 46.7% (7/15) of the participants in the intervention and control groups, respectively, had a higher average daily step count at the final visit (visit 3) than at the first visit (visit 1; *P*=.05).

**Table 2:** Changes in the average daily step count in the intervention and control groups during the study period.

				Intervention		Control		Difference (intervention–control)		*P* value
**Total study participants**										
	Number			25		25		0		
	**Average daily step count, mean (SD)**									
		Visit 1	11467 (5322)		11866 (5185)		–398 (5254)		.76^a^	
		Visit 2	11957 (5600)		11747 (4929)		210 (5282)		.88^a^	
		Visit 3	11550 (4060)		11345 (4432)		205 (4250)		.81^a^	
	**Change in the step count from visit 1, mean (SD)**									
		Visit 2	490 (4963)		–238 (2363)		728 (3913)		.53^a^	
		Visit 3	83 (5193)		–521 (4506)		603 (4862)		.50^a^	
	**Number of participants with an increase in the step count from visit 1, n (%)**									
		Visit 2	18 (72.0)		10 (42.0)				.03^b^	
		Visit 3	16 (64.0)		10 (40.0)				.09^b^	
**Subgroup of participants with a lower-than-average step count at visit 1 (<11667 steps/day)**										
	Number			14		15				
	**Average daily step count, mean (SD)**									
		Visit 1	7511 (2145)		8358 (1803)		–848 (1976)		.38^a^	
		Visit 2	9617 (4621)		8473 (2218)		1143 (3624)		.33^a^	
		Visit 3	10165 (2904)		9608 (3060)		557 (2986)		.67^a^	
	**Change in the step count from visit 1, mean (SD)**									
		Visit 2	2106 (3141)		160 (1986)		1946 (2628)		.08^a^	
		Visit 3	2655 (2581)		1250 (3233)		1405 (2937)		.19^a^	
	**Number of participants with an increase in the step count from visit 1, n (%)**									
		Visit 2	11 (78.6)		7 (50.0)				.12^b^	
		Visit 3	12 (85.7)		7 (46.7)				.05^b^	

^a^*P* value for comparing the intervention group with the control group calculated using a mixed model for repeated measures.

^b^*P* value for comparing the intervention group with the control group calculated using the chi-square test or Fisher exact test.

### Trajectories of Weekly Step Counts During the Study Period

We further performed an analysis with the linear mixed model with a random intercept and slope by using all 12 weeks of data to evaluate the impact of the intervention on weekly step counts (Figure S2 in Multimedia Appendix 2). In the intervention group, there was a significant increase of 1715 steps immediately after the intervention at 4 weeks (*P*=.02), but there was no significant difference compared to those in the control group. Overall, the linear mixed model with random intercepts and slopes revealed no significant interaction between time (weeks) and group (control vs intervention) during the study period (data were not shown).

### Changes in the Metabolic Parameters

Table 3 shows the changes in the metabolic parameters between the 2 groups. The intervention prevented the worsening of the metabolic parameters during the study period. The BMI, WC, FBS level, and HbA_1c_ level of the control group increased (*P*=.001, *P*=.01, *P*<.001, and *P*=.01 for BMI, WC, FBS level, and HbA_1c_ level, respectively), and most of the metabolic parameters did not change significantly in the intervention group. In particular, 44.4% (12/27) and 8% (2/25) of the participants in the intervention and control groups, respectively, had a decreased FBS level (*P*=.003; Table S2 in Multimedia Appendix 2). The proportion of the participants with MetS did not significantly change in either group.

Table 4 and Table S3 in Multimedia Appendix 2 show the changes in the metabolic parameters in the subgroup of the participants with a lower-than-average daily step count at baseline (<11,667 steps/day). Significant decreases in the WC and FBS levels were observed in the intervention group. Specifically, 57.1% (8/14) and 20% (3/15) of the participants in the intervention group and control groups, respectively, had a decreased WC (*P*=.04), whereas 50% (7/14) and 6.7% (1/15) of the participants in the intervention and control groups, respectively, had a decreased FBS level (*P*=.01).

To confirm the effects of walking on the metabolic parameters, we compared the metabolic parameters between the participants with and without an increased step count during the study period. We demonstrated that an increased daily step count had a beneficial effect on metabolic parameters (Table S4 in Multimedia Appendix 2). The proportion of the participants with decreased body weight, BMI, WC, FBS level, and HbA_1c_ level was significantly higher among those with an increased step count.

**Table 3:** Changes in the metabolic parameters in the intervention and control groups during the study period.

	Intervention (n=27)				Control (n=25)				Intervention vs control	
	V1^a^	V3^b^	*P* value^c^	V1^a^		V3^b^	*P* value^c^	*P* value for V1^d^		*P* value for V3-V1^e^
SBP^f^ (mm Hg), mean (SD)	117.7 (15.9)	113.3 (11.7)	.11	116.2 (10.4)		114.6 (8.0)	.44	.69		.42
DBP^g^ (mm Hg), mean (SD)	75.4 (11.3)	77.2 (9.4)	.45	72.9 (11.9)		73.2 (9.3)	.90	.44		.62
Body weight (kg), mean (SD)	56.8 (8.1)	57.2 (7.8)	.25	58.1 (6.2)		58.8 (6.4)	.15	.52		.55
BMI (kg/m^2^), mean (SD)	23.3 (2.8)	23.5 (2.7)	.12	23.0 (1.8)		23.5 (1.8)	.001	.66		.10
WC^h^ (cm), mean (SD)	79.9 (9.6)	81.1 (7.7)	.14	80.5 (5.6)		81.8 (5.8)	.01	.75		.99
FBS^i^ (mg/dL), mean (SD)	100.6 (12.5)	102.4 (14.4)	.35	97.2 (8.8)		103.6 (8.9)	<.001	.25		.001
HbA_1c_^j^ (%),mean (SD)	5.5 (0.7)	5.6 (0.6)	.13	5.3 (0.4)		5.4 (0.4)	.01	.26		.65
TG^k^ (mg/dL), median (IQR)	82 (62.0-103.5)	74 (59.0-104.5)	.67	75 (56.0-101.0)		95 (69.0-111.0)	.53	.35		.57
HDL-C^l^ (mg/dL), mean (SD)	58.2 (11.4)	56.7 (9.7)	.45	56.1 (11.0)		56.0 (8.3)	.90	.51		.57
Metabolic syndrome, n (%)	4 (14.8)	5 (18.5)	.66	3 (12.0)		3 (12.0)	>.99	.77		.54

^a^V1: visit 1.

^b^V3: visit 3.

^c^*P* values were calculated using the paired *t* test, the Wilcoxon signed rank test, or McNemar test.

^d^*P* value calculated using Student’s *t* test, the Wilcoxon rank-sum test, or the chi-square test.

^e^*P* value for the comparison of changes between groups calculated using Student’s *t* test or logistic regression for paired data.

^f^SBP: systolic blood pressure.

^g^DBP: diastolic blood pressure.

^h^WC: waist circumference.

^i^FBS: fasting blood sugar.

^j^HbA_1c_: glycated hemoglobin.

^k^TG: triglyceride.

^l^HDL-C: high-density-lipoprotein cholesterol.

**Table 4:** Proportion of participants demonstrating improvements in the metabolic parameters during the study period in the subgroup of participants with a lower-than-average daily step count at visit 1 (<11,667 steps/day).

	Intervention (n=14)	Control (n=15)	*P* value^a^
Decrease in SBP, n (%)^b^	7 (50.0)	10 (66.7)	.36
Decrease in DBP, n (%)^c^	5 (35.7)	7 (46.7)	.55
Decrease in body weight, n (%)	7 (50.0)	4 (26.7)	.20
Decrease in BMI, n (%)	7 (50.0)	3 (20.0)	.13
Decrease in WC, n (%)^d^	8 (57.1)	3 (20.0)	.04
Decrease in FBS, n (%)^e^	7 (50.0)	1 (6.7)	.01
Decrease in HbA_1c_, n (%)^f^	6 (42.9)	4 (26.7)	.45
Decrease in TG, n (%)^g^	7 (50.0)	7 (46.7)	.86
Increase in HDL-C, n (%)^h^	6 (42.9)	5 (33.3)	.60

^a^*P* value calculated using the chi-square test or Fisher exact test.

^b^SBP: systolic blood pressure.

^c^DBP: diastolic blood pressure.

^d^WC: waist circumference.

^e^FBS: fasting blood sugar.

^f^HbA_1c_: glycated hemoglobin.

^g^TG: triglyceride.

^h^HDL-C: high-density lipoprotein cholesterol.

## Discussion

This prospective, open-label, randomized pilot trial demonstrated that a wearable device-based physical activity intervention did not significantly increase the average daily step count in the North Korean refugees living in South Korea. Promoting a higher level of physical activity in the North Korean refugees who already walked >10,000 steps/day was challenging. However, the physical activity intervention using the wearable device and personalized exercise education was effective in the North Korean refugees with a lower-than-average daily step count. These individuals had a reduced WC and FBS level.

Refugees often have restricted access to health care services, which results in poor management of metabolic disease [10,19]. Financial barriers, a lack of security, and cultural impediments hinder refugees from accessing health care services [10,20,21]. Despite the increasing number of refugees diagnosed with chronic noncommunicable diseases, they are perceived as nonemergencies and are often ignored [21,22]. Health education and preventive health services are inadequate, necessitating the development of refugee health intervention programs. In this regard, self-monitoring of lifestyle-related parameters using wearable activity trackers may be useful for these vulnerable populations. However, our findings suggest that they are insufficient in improving the lifestyle and metabolic health of these populations. A possible explanation for the failure of the intervention is the low SES and high physical activity in the North Korean refugees. A previous meta-analysis demonstrated that digital interventions aimed at increasing physical activity showed significant efficacy in people with a high SES but not in those with a low SES [23]. In our study, most North Korean refugees had a low SES, and none had a high SES.

Furthermore, the proportion of participants in our study who engaged in vigorous physical activity was >20%. After using the wearable device, the average daily step count was >10,000. We investigated the occupations of the North Korean refugees to determine whether they required a high level of physical activity and found that 50% (26/52) of the participants had no jobs, and 19.2% (10/52) were students. Considering that most North Korean refugees had a low SES, they likely walked instead of driving a car, making it difficult to further increase their daily step count. Most of the remaining participants were blue-collar workers, which probably led to their high level of physical activity. Only 1 person had a sedentary job (administrative assistant).

Another primary concern is the cultural appropriateness of these interventions. Wearable devices and associated health programs often make implicit assumptions about users’ lifestyles and behaviors, which may not align with the unique experiences and cultural contexts of North Korean refugees [24]. This lack of cultural tailoring could limit the engagement of North Korean refugees with the intervention, thereby reducing its potential benefits. Additionally, privacy issues related to technology use could present barriers to the effective use of wearable devices in this population. The continuous monitoring and data recording associated with wearable devices can lead to apprehension and resistance among users, particularly in groups like North Korean refugees who may have historical reasons to mistrust authorities or systems [25]. These issues could negatively impact the consistent and correct use of the device, thereby limiting its potential impact on our results.

Previous studies have reported that depression could act as a barrier to performing physical activity [26]. Nearly half of the participants in our study were depressed, which is consistent with the finding of a previous study that the rate of depression among North Korean refugee participants is very high [27]_._ In this regard, the Fitbit device has some limitations in motivating patients with depression. This might explain our null results in a high proportion of North Korean refugee participants with depression. Further research should be conducted to determine whether this personalized exercise intervention with a wearable device can help patients recover from depression.

The North Korean Refugee Health in South Korea study aims to identify the health status of adult North Korean refugees living in South Korea. North Korean Refugee Health in South Korea study has been conducted since 2008 to evaluate the health outcomes of this population, and its results suggest that North Korean refugees have adapted to westernized South Korean society [28]. The prevalence rates of MetS in male (median age, 46 years) and female (median age, 44 years) North Korean refugees were comparable to those of the age- and sex-matched general population in South Korea [29]. However, excess weight gain after settling in South Korea is reportedly significantly associated with MetS in North Korean refugees [9,29]; this led us to perform this prospective randomized pilot study on physical activity and healthy lifestyles for optimal body weight maintenance in North Korean refugees. The prevalence of MetS in this study was relatively low (13.4%) than that in our previous studies [9,29]. We postulated that the duration of residence in South Korea was 2.8 years, which is shorter than that reported in previous studies, contributing to the low prevalence of MetS. Although we did not demonstrate a significant effect of the personalized exercise intervention with the wearable device on improving the daily step count, the pivotal finding of our study concerns the North Korean refugees with a lower-than-average daily step count. Among them, the effects of the intervention on improving the average daily step count were significant, and the intervention reduced metabolic parameters, including WC and FBS levels. Therefore, our study may serve as a cornerstone for large-scale prospective studies on North Korean refugees.

To the best of our knowledge, our study is the first prospective randomized pilot study to evaluate whether a personalized exercise intervention using a wearable device and education can increase the average daily step count and reduce the metabolic parameters in North Korean refugees. Nevertheless, this study had several limitations. First, a relatively small number of North Korean refugees were included in this prospective pilot study, which reduces the ability to generalize the results to the entire population of North Korean refugees living in South Korea and establish concrete conclusions. Because North Korean refugees have a poor SES, they usually have insufficient time to participate in clinical research studies. Therefore, recruiting and retaining North Korean refugees as participants in this study was challenging. Second, most participants already had a high daily step count and did not have MetS before study enrollment, making it difficult to show improvements in outcome measures due to the intervention. However, a subgroup analysis of the participants with a lower-than-average daily step count showed a beneficial effect of the intervention. Despite the small number of subjects in a subgroup, the change in metabolic parameters was statistically significant. Thus, a large-scale study that only enrolls North Korean refugees with inadequate physical activity and elevated cardio-metabolic risk is needed. Third, the 12-week study period may have been too short to fully elucidate the effects of the personalized physical activity educational intervention using the wearable device. Fourth, other factors than physical activity such as nutrition could influence metabolic parameters. Last, this study was conducted between 2020 and 2021, when social distancing was enforced because of the coronavirus disease pandemic. The coronavirus disease pandemic not only officially restricted the immigration of North Koreans to South Korea but also everyone’s activities, undoubtedly influencing the study’s results, particularly the average daily step count.

In this study, the wearable device–based physical activity intervention did not increase the average daily step count in the North Korean refugees, which reflects the difficulties in inducing behavioral changes in low SES populations. However, the intervention was effective in the North Korean refugees with a lower-than-average daily step count. Despite its limitations, this study has substantial clinical implications. The exploration of physical interventions in vulnerable refugee populations remains critical for understanding their unique characteristics. Moreover, this research aids in the development of effective strategies to ameliorate their health issues. Therefore, a long-term, large-scale study in an underserved population is warranted.

## Appendix

Multimedia Appendix 1 Trial protocol.

Multimedia Appendix 2 Supplementary figures and tables.

Multimedia Appendix 3 CONSORT-eHealth (V 1.6.1) checklist.

## Abbreviations

CES-D: Center for Epidemiological Studies Depression Scale
FBS: fasting blood sugar
HbA1c: glycated hemoglobin
HDL-C: high-density-lipoprotein cholesterol
IRB: institutional review board
MetS: metabolic syndrome
SES: socioeconomic status
TG: triglyceride
WC: waist circumference

## Data Availability

The data sets generated and analyzed during this study are available from the corresponding author upon reasonable request.
