# Supplementary material for: Effect of a Wearable Device–Based Physical Activity Intervention in North Korean Refugees: Pilot Randomized Controlled Trial
Source: J Med Internet Res. 2023 Jul 19;25:e45975. doi: 10.2196/45975 (PMC10398363; doi:10.2196/45975)
Supplement: Multimedia Appendix 1 [file jmir_v25i1e45975_app1.pdf]

1. Background

CRIS Registration Number	KCT0007999
Unique Protocol ID	2020AN0079
Public/Brief Title	The Effect of Wearable Device-based Physical Activity Intervention among North Korean Refugees
Scientific Title	Development of a personalized healthcare model for the management of lifestyle-related diseases in the North Korean defectors
Acronym	
MFDS Regulated Study	No
IND/IDE Protocol	
Registered at Other Registry	No
Healthcare Benefit Approval Status	Not applicable

2. Institutional Review Board / Ethics Committee

Board Approval Status	Submitted approval
Board Approval Number	2020AN0097
Approval Date	2020-02-27
Approval File	심사결과통보서.pdf
Institutional Review Board Name	Korea University Anam Hospital Institutional Review Board
Institutional Review Board Address	73, Inchon-ro, Seongbuk-gu, Seoul
Institutional Review Board Telephone	02-920-6566
Data Monitoring Committee	

Top
Bottom

3. Contact Details

- Contact Person for Principal Investigator / Scientific Queries

Name	Sin Gon Kim
Title	Professor
Telephone	+82-2-920-5890
Affiliation	Korea University Anam Hospital
Address	Division of Endocrinology and Metabolism, Korea University Anam Hospital,73 Goryeodae-ro, Seongbuk-gu, Seoul 02841, Korea

- Contact Person for Public Queries

Name	Ji Yoon Kim
Title	Clinical Associate Professor Equivalent
Telephone	+82-2-920-5767
Affiliation	Korea University Anam Hospital
Address	Division of Endocrinology and Metabolism, Korea University Anam Hospital,73 Goryeodae-ro, Seongbuk-gu, Seoul 02841, Korea

- Contact Person for Updating Information

Name	Ji Yoon Kim
Title	Clinical Associate Professor Equivalent
Telephone	+82-2-920-5767
Affiliation	Korea University Anam Hospital
Address	Division of Endocrinology and Metabolism, Korea University Anam Hospital,73 Goryeodae-ro, Seongbuk-gu, Seoul 02841, Korea

4. Status

Study Site	Single
Overall Recruitment Status	Completed
Date of First Enrollment	2020-06-13 Actual

Target Number of Participant	70
Primary Completion Date	2021-10-20 , Actual
Study Completion Date	2021-10-20 , Actual

- Recruitment Status by Participating Study Site 1

Name of Study	Korea University Anam Hospital
Recruitment Status	Completed
Date of First Enrollment	2020-06-13 ,

5. Source of Monetary / Material Support

- 1. Source of Monetary/Material Support

Organization Name	Ministry of Health & Welfare
Organization Type	Government
Project ID	HC19C0253

6. Sponsor Organization

- 1. Sponsor Organization

Organization Name	Korea University Anam Hospital
Organization Type	Medical Institute

7. Study Summary

Lay Summary	North Korean Refugees are often medically under-served and need proper management of metabolic disease. However, effective health promotion programs for North Korean Refugees have not been developed yet. This prospective, randomized controlled pilot study aims to evaluate the effects of digital health interventions using wearable device. This study aims to lay the groundwork for developing a personalized health promotion program for North Korean refugees.<br><br>North Korean Refugees aged 19-59 years are eligible and participants will be randomly assigned to the intervention and control groups. The intervention group will receive individual-level health counseling based on Fitbit data every four

	weeks, while the control group will wear the Fitbit without individual counselin g. Both groups will be followed up for 12 weeks. The change in average daily st eps recorded on wearable device and the change in metabolic parameters inclu ding body weight, waist circumference, triglycerides levels, high-density choles terol levels, blood pressure, blood glucose will be compared.

8. Study Design

Study Type		Interventional Study
Study Purpose		Prevention
Phase		Not applicable
Intervention Model		Parallel
Blinding/Masking		Open
Allocation		RCT
Intervention Type		Behavioral
Intervention Descripti on		The intervention group received individual-level health counseling based on Fit bit (wearable activity tracker) data every four weeks, while the control group w ore the Fitbit without individual counseling.
Number of Arms		2
Arm 1	Arm Lab el	Intervention group receiving individual-level health counseling based on Fitbit data
	Target N umber o f Partici pant	35
	Arm Typ e	Experimental
	Arm Des cription	For 12 weeks, the intervention group will receive individual-level health counsel ing every four weeks based on Fitbit data.

Arm 2	Arm Label	Control group wearing Fitbit without individual counseling
	Target Number of Participant	35
	Arm Type	No intervention
	Arm Description	For 12 weeks, the control group will wear Fitbit without individual-level health counseling.

9. Subject Eligibility

Condition(s)/Problem(s)		* (E00-E90)Endocrine, nutritional and metabolic diseases<br>(E88.8)Other specified metabolic disorders<br><br>Metabolic Syndrome
Rare Disease		No
Inclusion Criteria	Gender	Both
	Age	19Year~59Year
	Description	North Korean Refugees aged 19-59 years old using smartphones
Exclusion Criteria		Pregnancy
Healthy Volunteers		Yes

10. Outcome Measure(s)

Type of Primary Outcome	Efficacy

- Primary Outcome(s) 1

Outcome	The change in the average number of daily steps
Timepoint	At the end of the trial (after 12 weeks of trial)

- Secondary Outcome(s) 1

Outcome	The change in metabolic parameters including blood pressure, body weight, body mass index, waist circumference, fasting blood glucose, glycated hemoglobin, triglycerides, and high-density cholesterol levels

Timepoint	At the end of the trial (after 12 weeks of trial)

- Secondary Outcome(s) 2

Outcome	The change in the proportion of participants with metabolic syndrome
Timepoint	At the end of the trial (after 12 weeks of trial)

- Secondary Outcome(s) 3

Outcome	The change in lifestyle such as dietary habits
Timepoint	At the end of the trial (after 12 weeks of trial)

- Secondary Outcome(s) 4

Outcome	The change in moderate to vigorous physical activity
Timepoint	At the end of the trial (after 12 weeks of trial)

- Secondary Outcome(s) 5

Outcome	The change in sleep duration and heart rates recorded by wearable device
Timepoint	At the end of the trial (after 12 weeks of trial)

- Secondary Outcome(s) 6

Outcome	The change in depressed mood estimated by CES-D
Timepoint	At the end of the trial (after 12 weeks of trial)

11. Study Results and Publication

Result Registered	No

12. Sharing of Study Data(Deidentified Individual-Patient Data, IPD)

Sharing Statement	Yes
Time of Sharing	2023. 1
Way of Sharing	Available on Request<br>(k50367@korea.ac.kr)

목록으로 이동
