# Supplementary material for: Effect of a Wearable Device–Based Physical Activity Intervention in North Korean Refugees: Pilot Randomized Controlled Trial
Source: J Med Internet Res. 2023 Jul 19;25:e45975. doi: 10.2196/45975 (PMC10398363; doi:10.2196/45975)
Supplement: Multimedia Appendix 2 [file jmir_v25i1e45975_app2.docx]

**Multimedia Appendix 2**

**Effect of a Wearable Device–based Physical Activity Intervention in North Korean Refugees: Pilot Randomized Controlled Trial**

**Figure S1.** Flow diagram of the participant selection process used in the study.

**Figure S2.** Trajectories of weekly step counts during the study period.

**Table S1.** The specific occupations of the study participants (n=52).

**Table S2.** Proportion of participants demonstrating improvements in the metabolic parameters during the study period.

**Table S3.** Changes in the metabolic parameters during the study period for the subgroup of participants with a lower-than-average daily step count at visit 1 (<11667 steps/day).

**Table S4.** Changes in the metabolic parameters of the participants divided into subgroups according to the change in the average daily step count.

**Figure S1.** Flow diagram of the participant selection process used in the study.


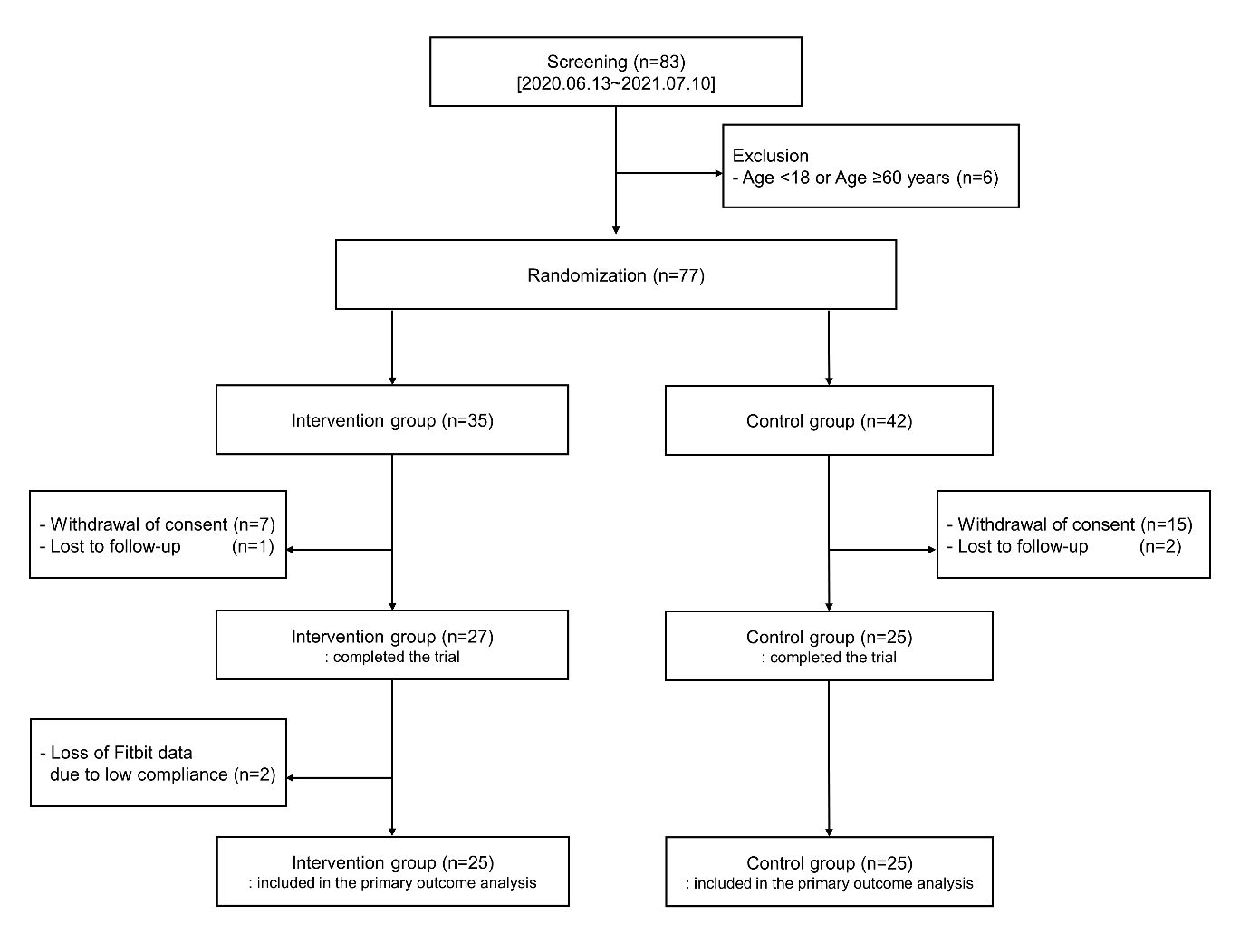


**Figure S2**. Trajectories of weekly step counts during the study period.


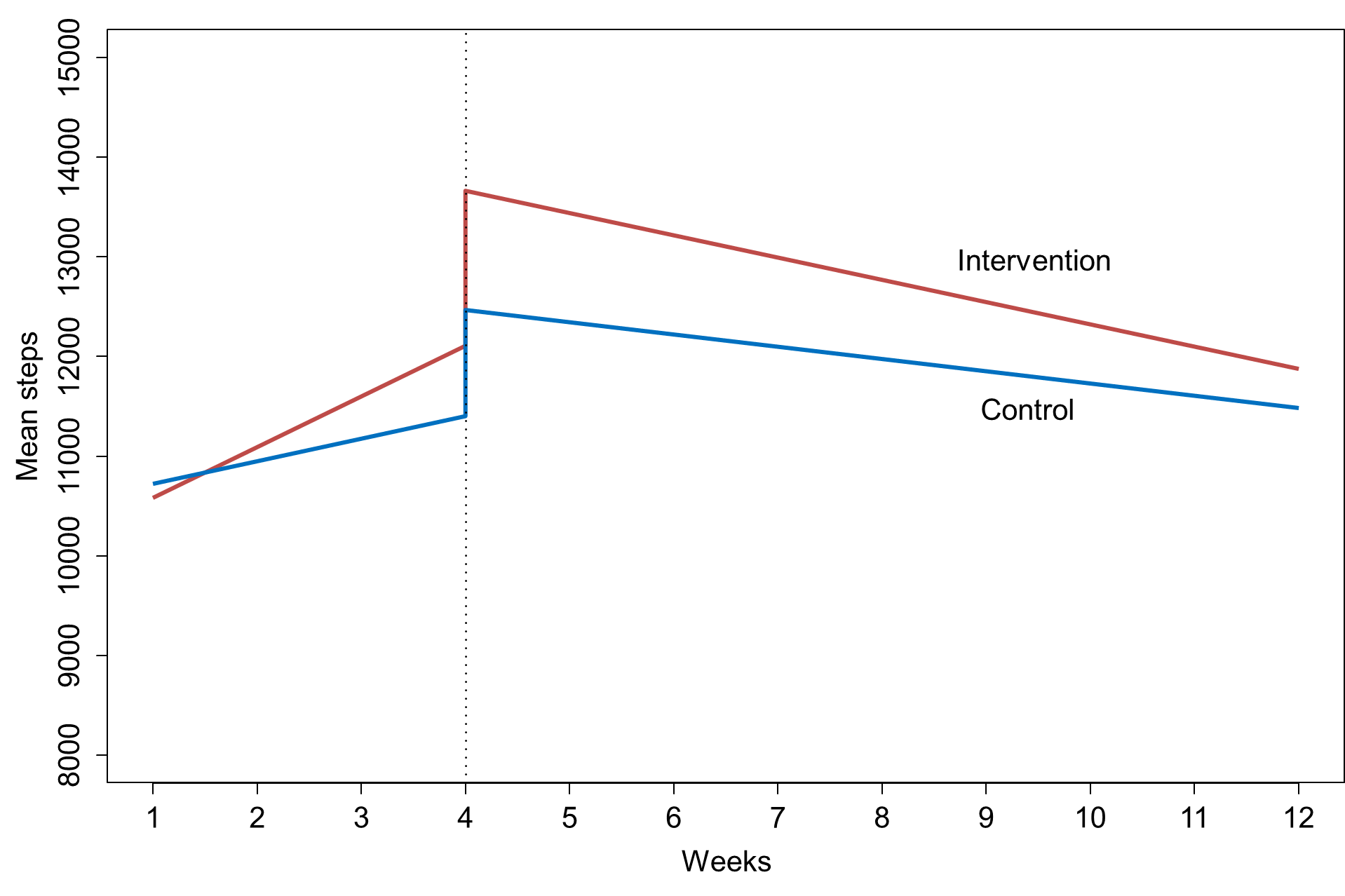


**Table S1.** The specific occupations of the study participants (n=52).

| Occupation | N (%) |
| --- | --- |
| Permanent worker | 5 (9.6) |
| Restaurant worker | 1 (1.9) |
| Machine worker | 1 (1.9) |
| Facility manager | 1 (1.9) |
| Seafood worker | 1 (1.9) |
| Temporary worker | 7 (13.5) |
| Facility manager | 2 (3.8) |
| Administrative assistant | 1 (1.9) |
| Product assembler | 1 (1.9) |
| Nursing assistant | 1 (1.9) |
| Salesperson | 1 (1.9) |
| Student | 10 (19.2) |
| Unemployed | 26 (50.0) |
| Others | 4 (7.7) |
| Cook | 1 (1.9) |
| Wireman | 1 (1.9) |
| Production worker | 1 (1.9) |
| Vendor | 1 (1.9) |

**Table S2.** Proportion of participants demonstrating improvements in the metabolic parameters during the study period.

|  | Intervention (n = 27) | Control (n = 25) | *P*^a^ |
| --- | --- | --- | --- |
| Decrease in SBP, n (%) | 15 (55.6) | 11 (44.0) | 0.974 |
| Decrease in DBP, n (%) | 12 (44.4) | 12 (48.0) | 0.797 |
| Decrease in body weight, n (%) | 9 (33.3) | 8 (32.0) | 0.918 |
| Decrease in BMI, n (%) | 10 (37.0) | 6 (24.0) | 0.309 |
| Decrease in WC, n (%) | 11 (40.7) | 6 (24.0) | 0.199 |
| Decrease in FBS, n (%) | 12 (44.4) | 2 (8.0) | 0.003 |
| Decrease in HbA1c, n (%) | 8 (29.6) | 7 (28.0) | 0.897 |
| Decrease in TG, n (%) | 13 (48.2) | 9 (36.0) | 0.376 |
| Increase in HDL-C, n (%) | 12 (44.4) | 11 (44.0) | 0.974 |

^a^*P*-value calculated using the chi-square test.

BMI, body mass index; DBP, diastolic blood pressure; FBS, fasting blood sugar; HbA1c, glycated hemoglobin; HDL-C, high-density lipoprotein cholesterol; SBP, systolic blood pressure; TG, triglyceride; WC, waist circumference.

**Table S3.** Changes in the metabolic parameters during the study period for the subgroup of participants with a lower-than-average daily step count at visit 1 (<11667 steps/day).

|  | Intervention  (n = 14) | | | Control (n = 15) | | | Intervention vs.  Control | |
| --- | --- | --- | --- | --- | --- | --- | --- | --- |
|  | V1 | V3 | *P*^a^ | V1 | V3 | *P*^a^ | *P*^b^ for V1 | *P*^c^ for V3–V1 |
| SBP (mmHg) | 115.5 ± 8.9 | 113.9 ± 6.8 | 0.534 | 117.8 ± 9.7 | 114.3 ± 6.8 | 0.193 | 0.514 | 0.618 |
| DBP (mmHg) | 75.3 ± 7.7 | 76.7 ± 9.6 | 0.540 | 72.8 ± 12.1 | 71.3 ± 9.2 | 0.512 | 0.518 | 0.366 |
| Body weight (kg) | 56.6 ± 6.8 | 56.3 ± 6.8 | 0.210 | 57.1 ± 6.6 | 57.9 ± 6.9 | 0.283 | 0.851 | 0.160 |
| BMI (kg/m^2^) | 22.8 ± 2.9 | 22.8 ± 2.8 | 0.623 | 22.7 ± 1.3 | 23.3 ± 1.3 | 0.001 | 0.850 | 0.001 |
| WC (cm) | 79.7 ± 6.9 | 79.4 ± 6.9 | 0.492 | 79.4 ± 5.4 | 80.7 ± 5.6 | 0.010 | 0.883 | 0.012 |
| FBS (mg/dL) | 96.4 ± 6.2 | 96.9 ± 6.2 | 0.833 | 96.3 ± 9.6 | 102.4 ± 9.5 | <0.001 | 0.958 | 0.023 |
| HbA1c (%) | 5.3 ± 0.2 | 5.5 ± 0.3 | 0.089 | 5.3 ± 0.5 | 5.4 ± 0.4 | 0.033 | 0.701 | 0.572 |
| TG (mg/dL) | 79.0  [65.0–104.0] | 73.5  [48.0–104.0] | 0.541 | 88.0  [66.0–165.0] | 96.0  [71.0–124.0] | 0.933 | 0.275 | 0.594 |
| HDL-C (mg/dL) | 57.1 ± 8.8 | 55.6 ± 9.5 | 0.520 | 55.6 ± 8.6 | 54.4 ± 7.1 | 0.373 | 0.654 | 0.927 |
| Metabolic  syndrome, n (%) | 2 (14.3) | 3 (21.4) | 0.564 | 1 (6.7) | 1 (6.7) | >0.999 | 0.598 | 0.312 |

Data are presented as the mean ± standard deviation, median [interquartile range], or number (%).

^a^*P*-value calculated using the paired t-test, the Wilcoxon signed-rank test, or McNemar's test.

^b^*P*-value calculated using Student's t-test, the Wilcoxon rank-sum test, or the chi-square test.

^c^*P*-value for the comparison of changes between the groups calculated using Student's t-test or logistic regression for paired data.

BMI, body mass index; DBP, diastolic blood pressure; FBS, fasting blood sugar; HbA1c, glycated hemoglobin; HDL-C, high-density lipoprotein cholesterol; SBP, systolic blood pressure; TG, triglyceride; V1, visit 1; V3, visit 3; WC, waist circumference.

**Table S4.** Changes in the metabolic parameters of the participants divided into subgroups according to the change in the average daily step count.

|  | Participants with an increased step count during the trial  (n = 26) | Participants without an increased step count during the trial  (n = 24) | *P*^a^ |
| --- | --- | --- | --- |
| Decrease in SBP, n (%) | 14 (53.9) | 13 (54.2) | 0.982 |
| Decrease in DBP, n (%) | 11 (42.3) | 12 (50.0) | 0.586 |
| Decrease in body weight, n (%) | 12 (46.2) | 4 (16.7) | 0.026 |
| Decrease in BMI, n (%) | 14 (53.9) | 1 (4.2) | <0.001 |
| Decrease in WC, n (%) | 13 (50.0) | 3 (12.5) | 0.005 |
| Decrease in FBS, n (%) | 11 (42.3) | 2 (8.3) | 0.006 |
| Decrease in HbA1c, n (%) | 11 (42.3) | 4 (16.7) | 0.048 |
| Decrease in TG, n (%) | 11 (42.3) | 11 (45.8) | 0.802 |
| Increase in HDL-C, n (%) | 11 (42.3) | 11 (45.8) | 0.802 |

^a^*P*-value calculated using the chi-square test.

BMI, body mass index; DBP, diastolic blood pressure; FBS, fasting blood sugar; HbA1c, glycated hemoglobin; HDL-C, high-density lipoprotein cholesterol; SBP, systolic blood pressure; TG, triglyceride; WC, waist circumference.
